# Monitoring online biomass with a capacitance sensor during scale-up of industrially relevant CHO cell culture fed-batch processes in single-use bioreactors

**DOI:** 10.1007/s00449-019-02216-4

**Published:** 2019-09-23

**Authors:** S. Metze, S. Ruhl, G. Greller, C. Grimm, J. Scholz

**Affiliations:** 1 Sartorius Stedim Biotech GmbH, August-Spindler-Str. 11, 37079 Göttingen, Germany; 2 Institut für Technische Chemie, Leibniz Universität Hannover, Callinstraße 5, 30559 Hannover, Germany

**Keywords:** Mammalian CHO cell culture, Process monitoring and control, PAT, Capacitance, Impedance, Scale-up

## Abstract

In 2004, the FDA published a guideline to implement process analytical technologies (PAT) in biopharmaceutical processes for process monitoring to gain process understanding and for the control of important process parameters. Viable cell concentration (VCC) is one of the most important key performance indicator (KPI) during mammalian cell cultivation processes. Commonly, this is measured offline. In this work, we demonstrated the comparability and scalability of linear regression models derived from online capacitance measurements. The linear regressions were used to predict the VCC and other familiar offline biomass indicators, like the viable cell volume (VCV) and the wet cell weight (WCW), in two different industrially relevant CHO cell culture processes (Process A and Process B). Therefore, different single-use bioreactor scales (50–2000 L) were used to prove feasibility and scalability of the in-line sensor integration. Coefficient of determinations of 0.79 for Process A and 0.99 for Process B for the WCW were achieved. The VCV was described with high coefficients of determination of 0.96 (Process A) and 0.98 (Process B), respectively. In agreement with other work from the literature, the VCC was only described within the exponential growth phase, but resulting in excellent coefficients of determination of 0.99 (Process A) and 0.96 (Process B), respectively. Monitoring these KPIs online using linear regression models appeared to be scale-independent, enabled deeper process understanding (e.g. here demonstrated in monitoring, the feeding profile) and showed the potential of this method for process control.

**Electronic supplementary material:**

The online version of this article (10.1007/s00449-019-02216-4) contains supplementary material, which is available to authorized users.

## Introduction

Scalability is a key aspect for biopharmaceutical companies to transfer a process that is producing an important protein or product from development stage to production scale. A bioprocess must reach production scale to be further considered for clinical trials in a pharmaceutical company and finally reach commercialization [1, 2]. A fast and reliable scale-up method enables faster development timelines and earlier entrance to the market with saving money and plant capacities. Therefore, it is desirable to have the ability to monitor all relevant parameters with the same measurement type in each process scale to keep the product quality and product quantity high and within GMP compliance [3–6]. Moreover, besides monitoring the process parameters, the demand for process control is strongly increasing [3, 7, 8]. The implementation of PAT supports online monitoring and real-time process control strategies. The application of PAT tools in biopharma processes is strongly suggested by the US Food and Drug Administration (FDA), underlined by the PAT initiative published in 2004. PAT enables real-time monitoring and control of critical process parameters (CPPs) that lead to consistent process performance and product quality [9–11].

The viable cell concentration (VCC) is one of the most important key performance indicator (KPI) during upstream technologies in mammalian cell culture [3]. However, often the VCC is measured by an offline method that stains dead cells with Trypan Blue and a cell count is done based on microscopic image analysis [12]. Online monitoring of biomass remains challenging as new technologies are more complex to calibrate or integrate into processes [13–16]. The limited samples per cultivation day as well as the time delay to respond to process changes are significant limitations of offline measurements that prevent efficient process monitoring and control of important CPPs. Several online methods to monitor the cell concentration of mammalian cell cultures have been investigated and developed in the last years (e.g. radio frequency impedance, Raman spectroscopy or near-infrared spectroscopy) [16–22].

One highly promising method to monitor online, the cell concentration is the radio frequency impedance measurement in the cell broth. The sensor principle is based on the polarization of the cells by applying a periodic alternating electric field to the system. Cells with an intact membrane can be seen as closed compartments in an aqueous system containing different ions, like salts or nutrient. Positive-charged ions will move towards the field and negative-charged ions will move in the contrary direction [13]. Both movements are limited by the plasma membrane being a barrier for both, the ions inside the membrane and the ions in the aqueous suspension. This effect generates a polarization at the poles of the cells that means that a charge separation takes place. These polarized cells change the relative permittivity of the liquid, and therefore the capacitance or impedance measurement changes. As only viable cells are polarizable, because of their non-disrupted membrane, capacitance measurements can be used to correlate viable cell correlations [23]. The capacitance measured in Farads describes the magnitude of the polarization that is induced by the field in the cell suspension [13]. By converting capacitance into absolute permittivity, the resulted values are normalized to the cell constant of the measurement arrangement, which corresponds mainly to the electrode geometry [24]. The absolute permittivity (*ε*) in pF/cm is calculated by the following: 

1 $$\varepsilon =C\times K$$

The measured capacitance can be used to calculate the relative permittivity (*ε*_r_) using:

2 $${\varepsilon }_{r}=C\times \left(\frac{K}{{\varepsilon }_{0}}\right)$$

The relative permittivity is dimensionless as it is relative to the electric constant (*ε*_0_) called the permittivity of free space that is equal to 8.854 × 10^–12^ F/m. *C* is measured in Farads and *K* is the cell constant measured in 1/m [24].

Dead cells or impurities in the cell culture broth are not detected by this method, because the outer cell membrane needs to be intact to enable polarization [13, 23]. One major difference of the measurement method compared to traditional VCC offline methods is that with increasing cell diameter, the cell polarization is different and the signal contribution of each cell increases. Therefore, in the death phase/apoptosis of a mammalian CHO cell culture, where the cell diameter is increasing, the permittivity signal does not correlate with the viable cell concentration anymore [25, 26]. There is, however, the discussion among the scientific community for strong evidence that permittivity is more robust than VCC for certain applications. Measuring cell mass or online viable cell volume (VCV) can be better for automated feeding strategies, because larger cells usually demand more nutrients and that is not accounted for by the traditionally used cell count [27].

Capacitance sensors were frequently used in the past to monitor different cell lines in bioprocesses (e.g. mammalian cells, insect cells or microcarrier cultures) [28–30]. Many times the signal was treated and correlated to different process parameter such as the VCV, the VCC, or the total cell count [13, 27, 31]. Besides linear regression to correlate the permittivity with selected parameters, linear mixed effects (LME) models or multiple frequency measurement analysis via more complex mathematical modeling such as Cole–Cole modeling or Partial Least Square Regression (PLS) can be done to achieve online monitoring of important parameters [13, 27, 32–35]. However, measuring the capacitance at one frequency offers several advantages: The method is easy to implement, and offers a fast measurement principle which is important, especially in rocking motion bioreactors. Additionally, no sophisticated data processing is needed. Linear regressions were reported to deliver satisfying results for many applications and parameters [26].

Even though, research was done on the field of capacitance measurements, this technique is not yet a standard measurement principle for process monitoring or control in industry or research departments dealing with mammalian cell cultivation.

In this work, the scalability and transferability of the capacitance measurement principle from small scale to large scale and single-use bioreactors up to 2000 L were investigated. Besides the scalability, the comparability and prediction ability of KPIs were studied using single-use bioreactors. Single-use bioreactors raised high attention in the last years enabling fast turnover times, reducing costs, and providing dynamic plant capacities [36–39]. The work investigates the use of a capacitance sensors from process development to production scales. A fully scalable process in single-use bioreactors with monitoring KPIs and CPP control by PAT tools can significantly optimize new product development and fast market entrance for biopharmaceutical companies. The presented studies analyze the possibilities to provide a scale-independent linear regression model for the VCC, the VCV, and the wet cell weight (WCW) based on online capacitance measurements. Traditionally VCC is the main parameter used to monitor the cell growth of a mammalian cell culture. WCW is important for downstream processing and the correct selection of devices to purify the product. VCV as a KPI is increasing in interest and a correlation between permittivity and VCV is expected to show best results based on the measurement principle [27]. Moreover, the work investigates applications for two different cell lines to prove, whether the approach can be applied on a large variety of mammalian CHO cell cultures.

## Materials and methods

### Cell lines and media

Two DG44 CHO cell lines expressing different monoclonal antibodies were used in this study (Cell line A and cell line B). Seed medium (SM), basal medium for production (PM), and two different feeds: feed medium A (FMA) and feed medium B (FMB) were used for all studies (Sartorius Stedim Cellca GmbH). All media and feeds were chemically defined.

### Seed culture for Process A and Process B

The seed culture was performed similar for both processes. A cryovial containing 1 mL CHO suspension (passage 8) at a concentration of 30 million cells/mL was thawed and transferred into a 15 mL Falcon^®^ tube (Sarstedt) with 10 mL thermalized (36.8 °C) seed medium. This suspension was centrifuged (Centrifuge 3-30 K, Sigma) at 190*g* at room temperature for 3 min to remove all components of the freezing medium. After decanting the supernatant, the pellet was resuspended with 10 mL pre-warmed seed medium and transferred into a 500 mL Erlenmeyer flask (Corning) filled with 150 mL pre-warmed seed medium. The shake flask was incubated in an incubation shaker (Certomat CTplus, Sartorius Stedim Biotech) at 36.8 °C and 7.5% pCO_2_ with a shaking rate of 120 rpm and 85% humidity. Cells were passaged five times every 3–4 days until inoculation of the production culture was done.

To increase the volume of the pre-culture for the large bioreactor scales (50–500 L) in the main stage, the last pre-culture steps were moved from shake flasks to rocking motion bioreactors (BIOSTAT^®^ RM 20/50, Sartorius Stedim Biotech). The temperature set point was chosen at 36.8 °C and the pH was controlled at 7.1 (for Process A) and 7.15 (for Process B) through CO_2_ sparging. The rocking rate of each bioreactor was set to 30 rpm with an angle of 10° and dissolved oxygen (DO) was controlled at 60%.

The largest main culture bioreactors (1000–2000 L) were inoculated with a pre-culture in a stirred-tank bioreactor (BIOSTAT^®^ STR 200/500, Sartorius Stedim Biotech). The temperature set point was chosen at 36.8 °C and the pH was controlled at 7.1 (for Process A) and 7.15 (for Process B) through CO_2_ sparging. The stir speed of each bioreactor was set to 120 rpm (200 L) or 96 rpm (500 L), respectively. The DO was controlled at 60%.

### Main culture for Process A

The main culture was inoculated with 0.3 million cells/mL. The main process was conducted at 36.8 °C ± 0.05 °C. The pH was set to 7.0 and controlled by the addition of CO_2_. Once in a day the pH was measured offline (see 2.6 Offline Analytics) and compared to the online measurement. If the result deviated by more than 0.05, the online sensor was recalibrated.

The set point for DO was set to 60%. For inoculation, N_2_ gas was sparged to adjust the DO to 60%. The initial gassing rates were adjusted using N_2_, air, and oxygen to keep a k_L_a of 7.9 1/h (based on previous process engineering characterizations). The bioreactors were stirred according to their scale with 162 rpm (50 L), 121 rpm (200 L), 96 rpm (500 L), 86 rpm (1000 L), and 70 rpm (2000 L), respectively. The stir speed was adjusted according to the scale-up strategy in keeping the k_L_a of 7.9 1/h constant in all scales. On the day of inoculation, antifoam (2% Antifoam C Emulsion, Sigma-Aldrich^®^) was added (0.001% of the cell suspension volume). During the cultivation, antifoam was added manually by the operator depending on the foam level.

Starting from inoculation day, which was day 0, the cultivation lasted for 12 days. FMA and FMB were supplied from day 3 in a ratio of 10:1 (FMA: FMB) with a feed amount of FMA of 42 g/L start volume/day and FMB, accordingly. The volume changes inside the bioreactor based on the feeding strategy are demonstrated in Fig. S1.

Starting from day 5, depending on when the glucose level dropped below 5 g/L, glucose was added to the cell broth as a bolus to hold the glucose concentration at 5 g/L.

### Main culture for Process B

Similar to Process A, the main culture was inoculated with 0.3 million cells/mL and the main process was conducted at 36.8 °C ± 0.05 °C. The pH was set to 7.15 and controlled by the addition of CO_2_. If the offline pH deviated by more than 0.05 the online sensor was recalibrated on a daily basis.

The set point for DO was set to 60%. For inoculation, N_2_ gas was sparged to adjust the DO to 60%. The initial gassing rates were adjusted using N_2_, air, and oxygen to keep a k_L_a of 7.9 1/h. The bioreactors were stirred according to their scale with 170 rpm (50 L), 124 rpm (200 L), and 86 rpm (1000 L), respectively. The stir speed was adjusted according to the scale-up strategy in keeping the k_L_a of 7.9 1/h constant in all scales. On the day of inoculation, antifoam was added (0.001% of the cell suspension volume). During the cultivation, antifoam was added manually by the operator depending on the foam level.

Starting from inoculation day, which was day 0, the cultivation lasted for 17 days. FMA and FMB were supplied from day 3 in a ratio of 10:1 (FMA: FMB) with a feed amount of FMA of 43.2 g/L start volume/day and FMB accordingly. Equally to Process A, the volume changes inside the bioreactor based on the feeding can be seen in Fig. S1.

Starting from day 5, depending on when the glucose level dropped below 5 g/L, glucose was added to the cell broth as a bolus to hold the glucose concentration at 5 g/L.

### Online capacitance measurements

Capacitance measurements were conducted with an impedance probe (BioPAT^®^ ViaMass, Sartorius Stedim Biotech for single-use applications and a Futura 12 mm Probe, Aber Instruments Ltd for multi-use applications). The sensor was set to cell culture mode measuring at one single frequency at 580 kHz and a filter over 30 values was used. The probe was either directly connected to a BioPAT^®^ DCU (Sartorius Stedim Biotech), BioPAT^®^ MFCS/win was used for data acquisition and the data were finally stored in an internal database or the probe was connected with a connection hub (Futura Connect, Aber Instruments Ltd) to a PC. On the PC it was processed by the Futura Tool^®^ software (Aber Instruments Ltd.). The data were stored as .csv format and imported to an Excel^®^ file (Microsoft Cooperation) for further treatment.

### Offline analytics

The viable cell concentration indicating the amount of viable cells in the cultivation and the viability of cells according to the total cell concentration, as well as the average cell diameter, were analyzed with the Trypan Blue Assay based Cedex HiRes Cell Counter and Analyzer system (Roche). The pH and the glucose concentration were measured offline in a blood gas analyzer (ABL800 Basic, Radiometer).

For the WCW measurement, 5 mL of the cell broth was transferred into a 15 mL Falcon^®^ tube (Sarstedt). The cell suspension was centrifuged (Centrifuge 3–30 K, Sigma) at 5000*g* at room temperature for 5 min. After centrifugation the media was removed and the pellet was weighed (Genius, Sartorius AG). The WCW was calculated according to Eq. 3, with w_Pellet_ indicating the weight of the tube together with the pellet and w_Tube_, the empty weight of the falcon before adding the cell suspension. The sample volume described the amount of cell broth that was added to each tube before centrifugation.

3 $$\mathrm{W}\mathrm{C}\mathrm{W}=\frac{{(w}_{\mathrm{P}\mathrm{e}\mathrm{l}\mathrm{l}\mathrm{e}\mathrm{t}}- {w}_{\mathrm{T}\mathrm{u}\mathrm{b}\mathrm{e}) }}{\mathrm{S}\mathrm{a}\mathrm{m}\mathrm{p}\mathrm{l}\mathrm{e}\mathrm{ }\mathrm{v}\mathrm{o}\mathrm{l}\mathrm{u}\mathrm{m}\mathrm{e}}$$

The VCV was calculated based on the offline VCC, the diameter measured in the Cedex and the equation of a sphere indicating the cell shape according to:

4 $$\mathrm{V}\mathrm{C}\mathrm{V}= \frac{4}{3} \pi {\left(\frac{\mathrm{C}\mathrm{e}\mathrm{l}\mathrm{l}\mathrm{ }\mathrm{d}\mathrm{i}\mathrm{a}\mathrm{m}\mathrm{e}\mathrm{t}\mathrm{e}\mathrm{r}}{2}\right)}^{3}\times \mathrm{V}\mathrm{C}\mathrm{C}$$

## Results and discussion

### Comparison of biomass related online and offline measurements in the smallest (50 L) and the largest (2000 L) bioreactor scale

In the following experiments, the suitability of online capacitance measurements for biomass monitoring during scale-up was investigated.

**Fig. 1:**
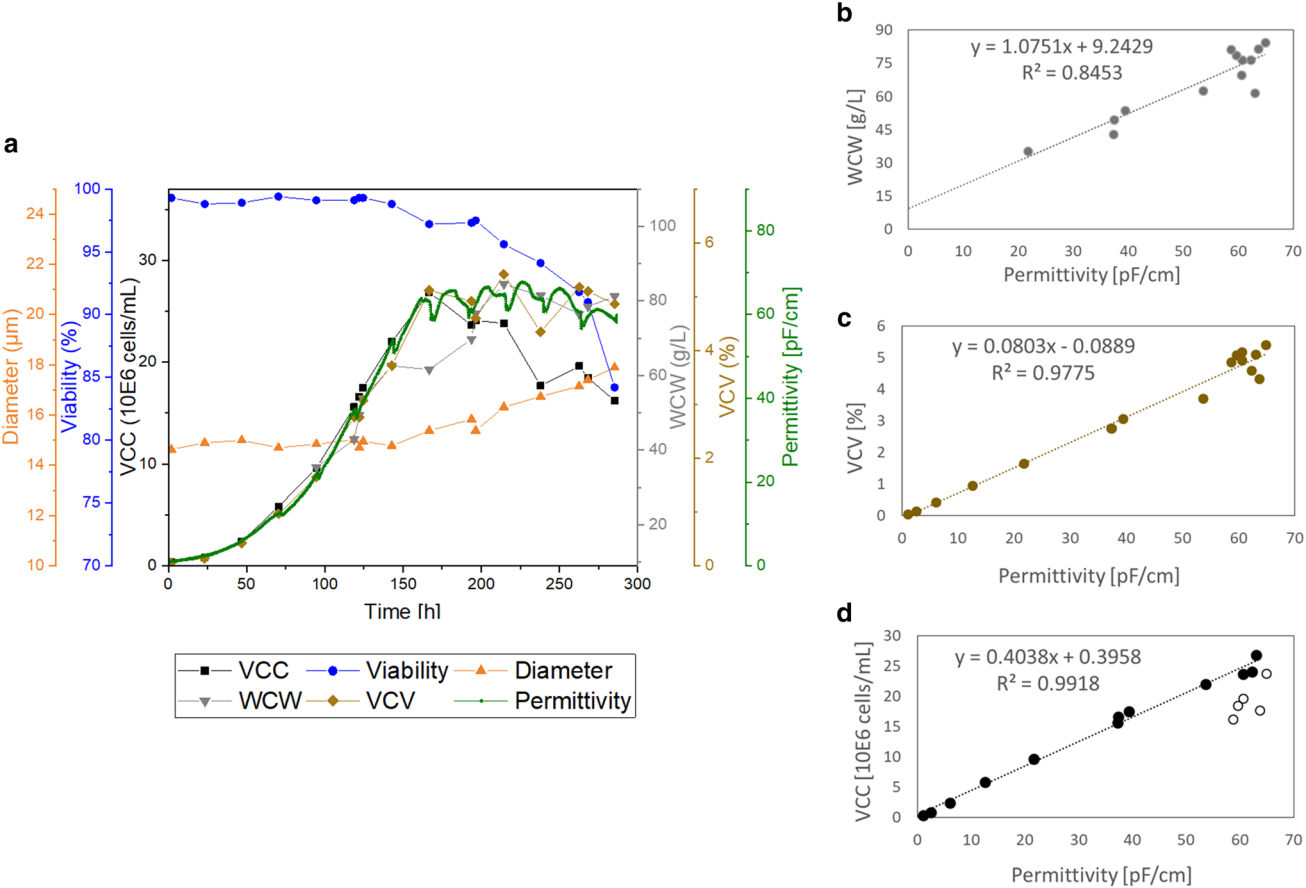
Overall cultivation results of the STR50 cultivation of process A. Top **a**: time-dependent cultivation results for cell diameter, cell viability, viable cell concentration (VCC), wet cell weight (WCW), viable cell volume (VCV), and online permittivity signal. Bottom: correlation of the permittivity signal with WCW (**b**), VCV (**c**), and VCC (**d**). Full circles in **d** represent the data points incorporated into the linear regression model (up to peak VCC) and empty circles represent the values excluded from the linear regression (stationary and apoptotic cell growth phase)

**Fig. 2:**
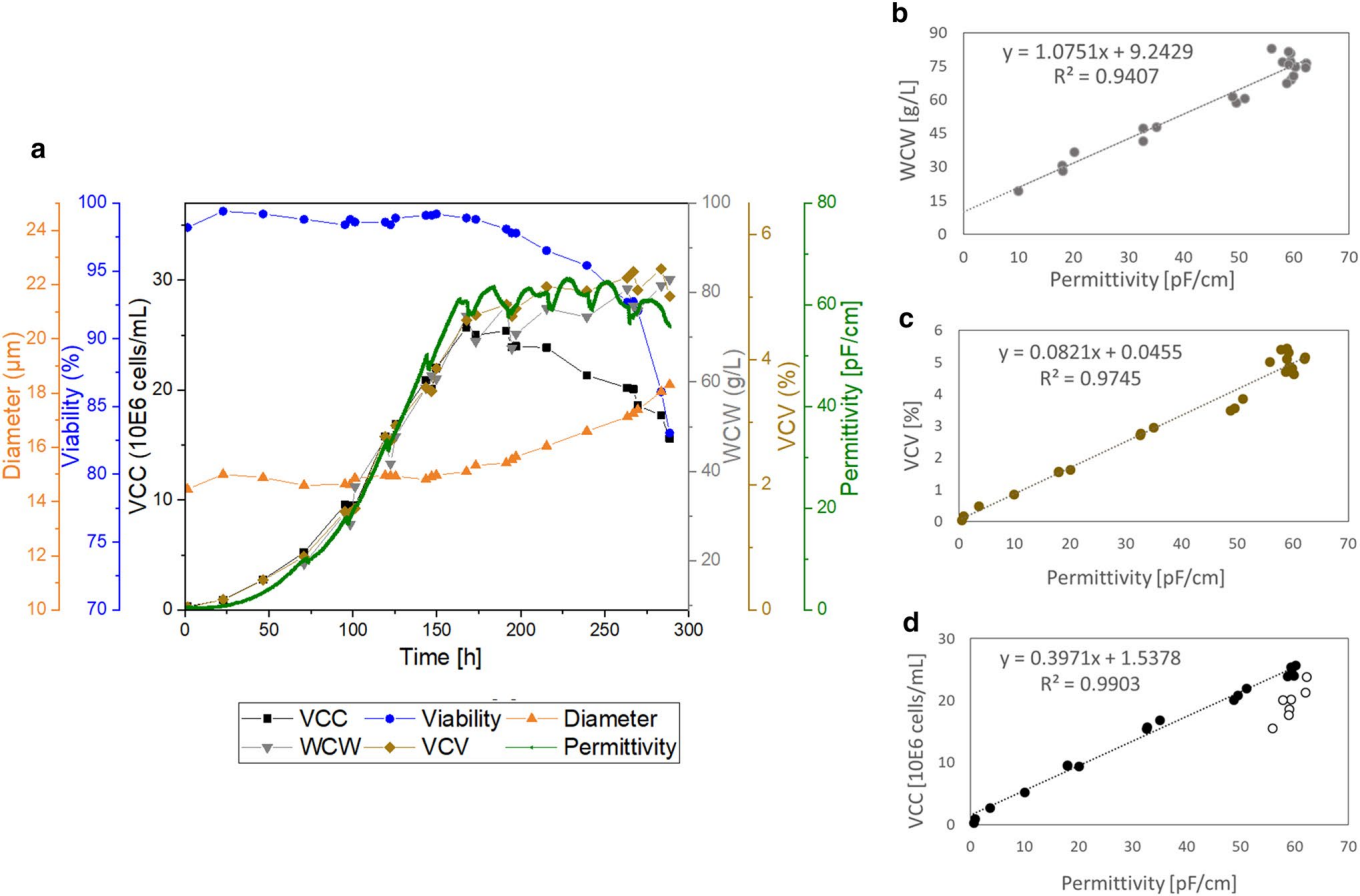
Overall cultivation results of the STR2000 cultivation of process A. Top **a**: time-dependent cultivation results for cell diameter, cell viability, viable cell concentration (VCC), wet cell weight (WCW), viable cell volume (VCV), and online permittivity signal. Bottom: correlation of the permittivity signal with WCW (**b**), VCV (**c**), and VCC (**d**). Full circles in **d** represent the data points incorporated into the linear regression model (up to peak VCC) and empty circles represent the values excluded from the linear regression (stationary and apoptotic cell growth phase)

As a first step, the online signal was compared and correlated to three different offline methods for biomass estimations in the smallest investigated bioreactor size of 50 L (Fig. 1) and the largest size of 2000 L (Fig. 2). The VCC was measured by Trypan Blue Assay in a semi-automated system. The same system detected the diameter so that by calculation (Eq. 3), the VCV was determined. Finally, the WCW was achieved in a fully manual assay according to Eq. 3. To better understand the deviations that are in an acceptable range for the correlations, the errors of each method are listed in Table 1.

**Table 1:** List of errors for measurement methods that are presented in the studies

Method	Error expectations (%)	Reference for error expectations
Wet cell wet	5–25	Error range detected during performed measurements
Viable cell concentration	10	Typical detected error in all performed measurements is not better than the stated error and in agreement with reports in the literature [22]
Viable cell volume	16	Error propagation based on expected Viable Cell Concentration error and 2% estimated error in diameter detection that was detected during the presented and previous measurements

For both scales the offline data points that are describing the biomass in the process are showing the same tendencies and results as the online permittivity signal depending on the culture time (Figs. 1a, 2a). As long as the cells were in the exponential growth phase, the VCC, the VCV, the WCW, and the permittivity are overlapping and correlating with each other. An advantage of the capacitance measurement compared to the offline references can be seen immediately in both figures (Figs. 1a, 2a). The dips starting from 72 h on reflect the feeding in the fed-batch processes. Thus, in the permittivity signal, it is possible to monitor the feeding online. Information about the feeding profile and the effect on the cells can be detected online with the sensor integration.

In Fig. 1b–d and Fig. 2b–d, a linear correlation used to describe the relationship of the online permittivity signal with the corresponding offline parameters and the coefficient of determination was investigated.

The WCW is a manual assay with a high expected error of up to 25% in the measurement principle itself (see Table 1). In the smallest scale (Fig. 1b), this resulted in a coefficient of determination of 84.5%, which is the lowest detected coefficient of determination for the investigated parameters. For the 2000 L scale, the correlation between WCW and permittivity improved to 94.1%. This result from the 2000 L run shows that the online permittivity can describe the WCW within an acceptable range. The deviation, especially in the 50 L scale, was most likely caused by manual operations, changes in the operator or wrong execution of the reference method. Furthermore, there were few data points taken at low WCW in the beginning of each cultivation and the data points at the end of the cultivations showed a large scatter. A straight line through the origin was expected with WCW measurements. However, due to the non-symmetric point’s distribution, the linear regression model resulted in high *y* axis intercepts in both scales. Therefore, the correlations of the WCW shows relatively high errors at small WCWs. Using the online permittivity signal to estimate the WCW instead of offline samples could even improve the WCW determination throughout the complete cultivation.

Referring to the correlation of online permittivity and VCV in both scales, the coefficient of determination was 97.8% (Figs. 1c, 2c). Therefore, it can be concluded that the online sensor is capable to describe the VCV in a highly accurate manner.

As the permittivity signal has deficits in describing the VCC in the death phase, differences after the peak cell concentration were expected. As described in the introduction, the permittivity is increasing with higher cell concentration as well as higher cell diameters. Indeed, deviations in the data points of the 50 L and the 2000 L scale were detected with increasing diameter and decreasing viability (Figs. 1a, 2a).

Therefore, the correlation of the VCC and the online signal was based on a selected set of data points until the end of the exponential growth phase (Figs. 1d, 2d, full circles). The values in the death phase were not considered (Figs. 1d, 2d, empty circles). For the WCW and the VCV, the complete process duration was included in the correlations.

The VCC in the exponential cell growth could be monitored by the online sensor with a coefficient of determination of 99.2% for the 50 L scale and 99.0% for the 2000 L scale. Compared to the 10% measurement errors that exist in the offline reference (Table 1), the permittivity signal is robust and reliable. Therefore, the sensor is suitable for both bioreactor scales, the small bioreactor of 50 L and the large bioreactor of 2000 L. The permittivity signal can successfully monitor the VCC during the exponential growth phase of the cells.

These results give a first indication for the suitability of an online capacitance sensor to monitor cell growth in the given cell culture process during scale-up experiments. In the following, the transfer to other scales (200 L, 500 L, and 1000 L) as well as other cell clones was tested to achieve a broader correlation that predicts VCC, VCV, and WCW based on the online permittivity signal.

### Investigation of scalability of single-use bioreactors based on online permittivity trajectories

After successful demonstration of the correlation of the online permittivity signal to different biomass related process parameters in the smallest and largest bioreactor size, the trajectory of the permittivity signal should be analyzed through all involved bioreactor scales (50 L, 200 L, 500 L, 1000 L, and 2000 L).

**Fig. 3:**
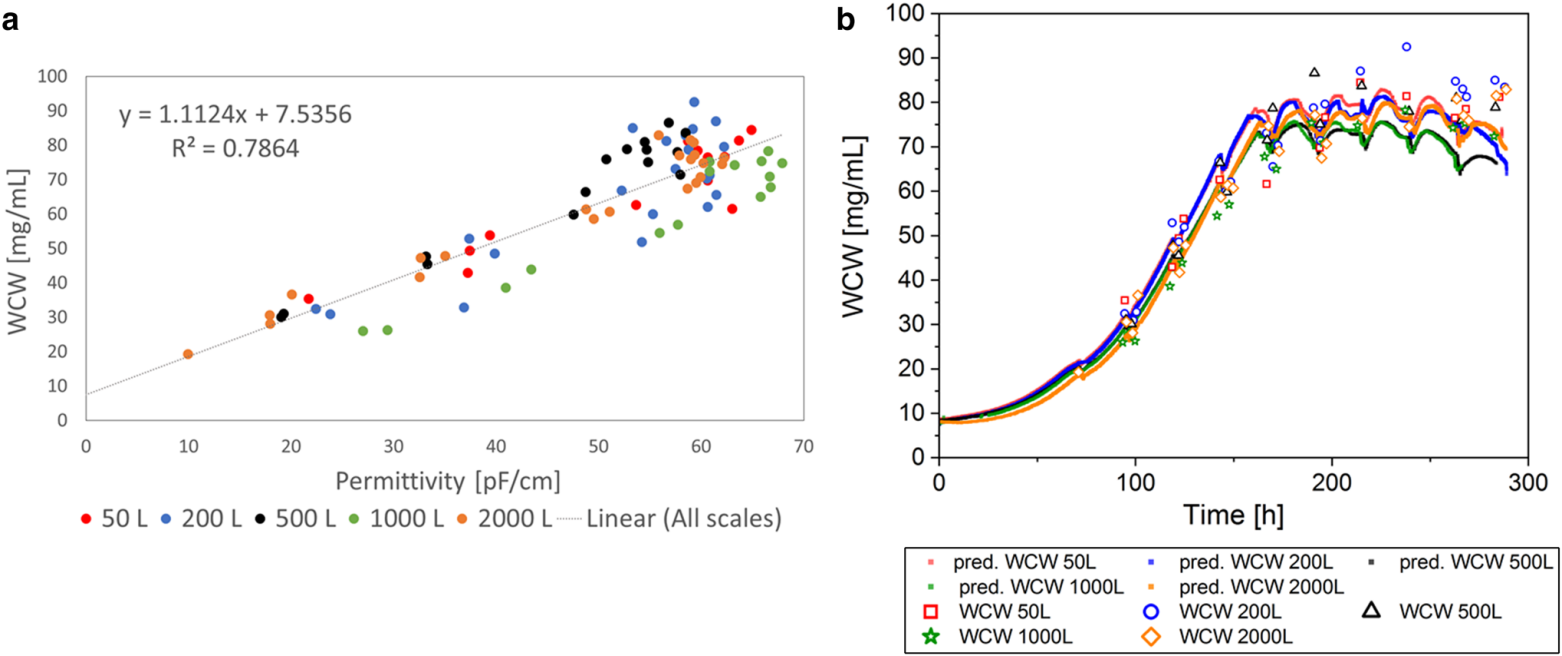
Linear regression model of permittivity and wet cell weight (WCW) for Process A including all cultivations from 50 L up to 2000 L bioreactor volume (**a**). Predictions (pred.) based on the online permittivity signal for all bioreactor scales using the equation from the linear regression (**b**)

**Fig. 4:**
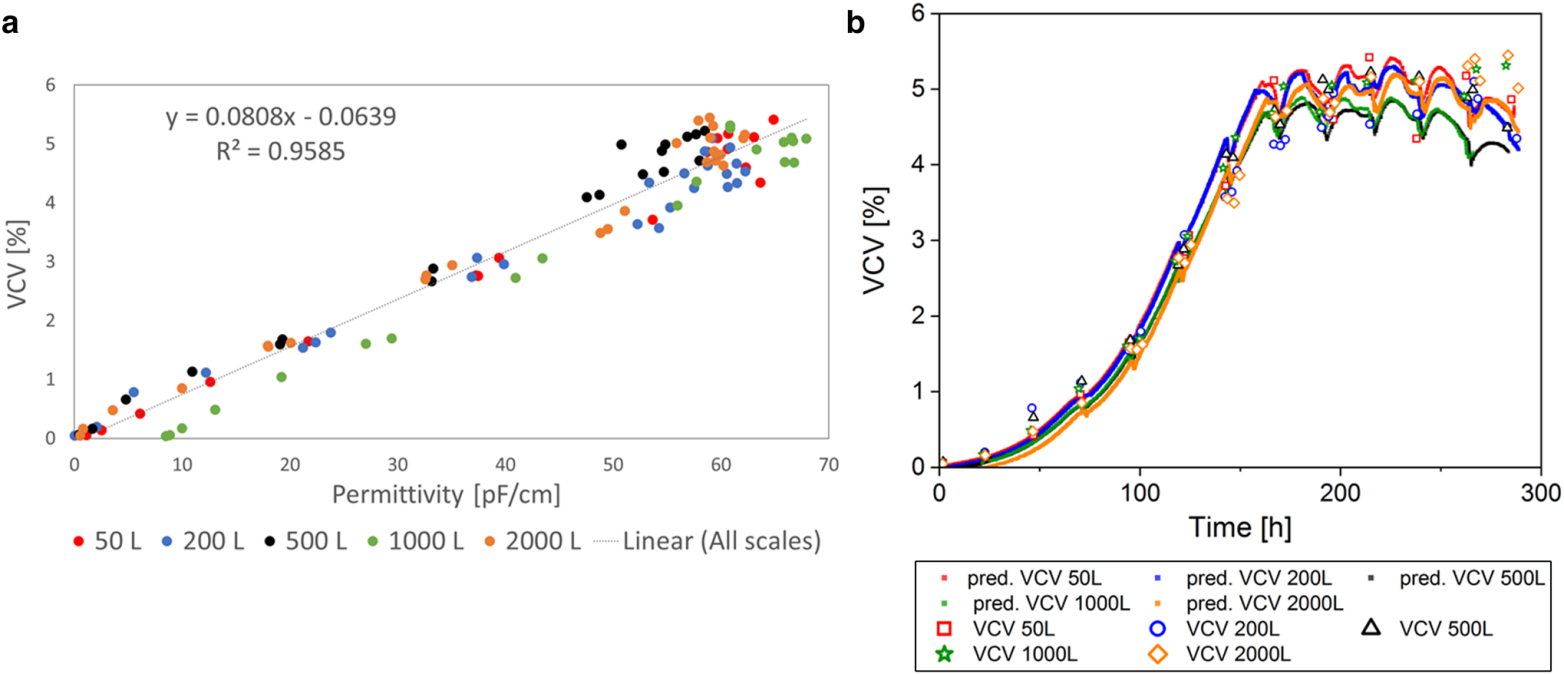
Linear regression model of permittivity and viable cell volume (VCV) for Process A, including all cultivations from 50 L up to 2000 L bioreactor volume (**a**). Predictions (pred.) based on the online permittivity signal for all bioreactor scales using the equation from the linear regression (**b**)

**Fig. 5:**
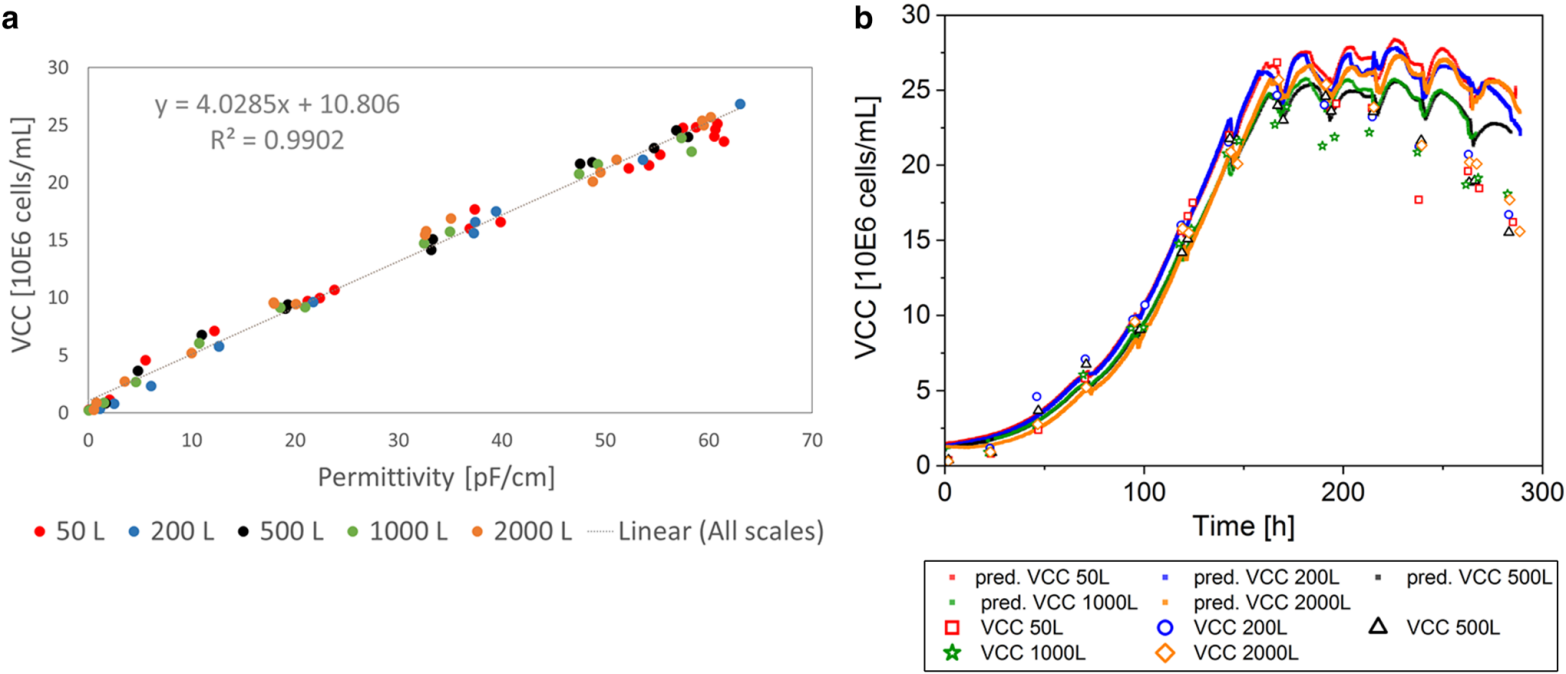
Linear regression model of permittivity and viable cell concentration (VCC) for Process A, including all cultivations from 50 L up to 2000 L bioreactor volume (**a**). VCC only considered up to peak VCC for linear regression. Predictions (pred.) based on the online permittivity signal for all bioreactor scales using the equation from the linear regression (**b**)

To investigate the scalability of the permittivity signal, the different single-use bioreactor cultivation data sets were combined and included in each linear correlation for WCW, VCV, and VCC (Figs. 3, 4, 5). Within the correlation to each offline value of the parameters, the corresponding online value was selected and included in the linear model. The linear regression model was used to predict the respective values based on the complete online permittivity data sets. In the linear regression, each batch was colored individually to investigate the impact of a specific bioreactor scale or cultivation on the regression model (Figs. 3a, 4, 5a). However, there was no systematic difference in each regression model between scales and cultivations detected. This result indicates a robust and scalable process that enables a scale-independent linear model to predict the WCW, VCV, and the VCC (VCC only until end of exponential growth phase).

As described above and in Table 1, the WCW is a manual assay, which can have a large error up to 25% based on the method itself and influence of the operator. Based on the theory, a good correlation between permittivity that represents the volume of viable cells and WCW should be given. Surprisingly, combining all scales in one linear model, led to a relative low coefficient of determination of 78.6% (Fig. 3a). The measurement values were highly scattered and only few data points were available for small WCWs for all processes. Therefore, the available data set was insufficient for a representative regression. The highest deviation could be seen in the apoptotic cell status (Fig. 3b). This result might indicate strong manual deviations depending on the operator and frequently changed operators over the process time. As a result of the poor regression, there was no straight line through the origin detected in contrast to previous expectations. In summary, the low coefficient of determination can be seen as a result of an inconsistent offline method with errors up to 25% between each measurement and a very inhomogeneous sample distribution over the measurement range (Table 1). Even though much better results were expected, considering the error of the offline method for each measurement point, the online signal might be used to predict the WCW during scale-up in the future.

The correlation of the VCV and the online permittivity signal had a coefficient of determination of 95.9% (Fig. 4a) in a combined linear model for all scales. Especially, in the exponential growth phase, the VCV was described highly accurate (Fig. 4b), whereas in the death phase, the predicted values deviated as seen in the WCW. Remembering that the offline method results in errors of up to 16% (Table 1), the resulted coefficient of determination implies a stable prediction and can be used for online monitoring of the VCV in different bioreactor scales.

The VCC was correlated only for the exponential growth phase, as described previously. In the exponential growth phase, the linear model for all scales described 99.0% of the data points (Fig. 5a). The prediction of the VCC in the exponential growth phase based on the online signal, described the offline data reference precisely (Fig. 5b). The online signal was not capable to predict the VCC in the death phase of the cells. Nevertheless, the scalability of the linear model and the transfer from scale to scale was working in the exponential growth phase. Therefore, the online signal can be a useful tool to support process scale-up. Even though limitations of the permittivity signal for the VCC prediction in the death phase could be detected, real time process monitoring of the VCC during the exponential growth phase was possible. By including the cell diameter into the calculations as it is shown in the VCV, an accurate linear model to describe all bioreactors was given. The real-time information based on the permittivity signal enables to monitor and control the bioprocess, as process deviations can be immediately recognized.

### Proof of concept with different CHO cell line and cross verification

**Fig. 6:**
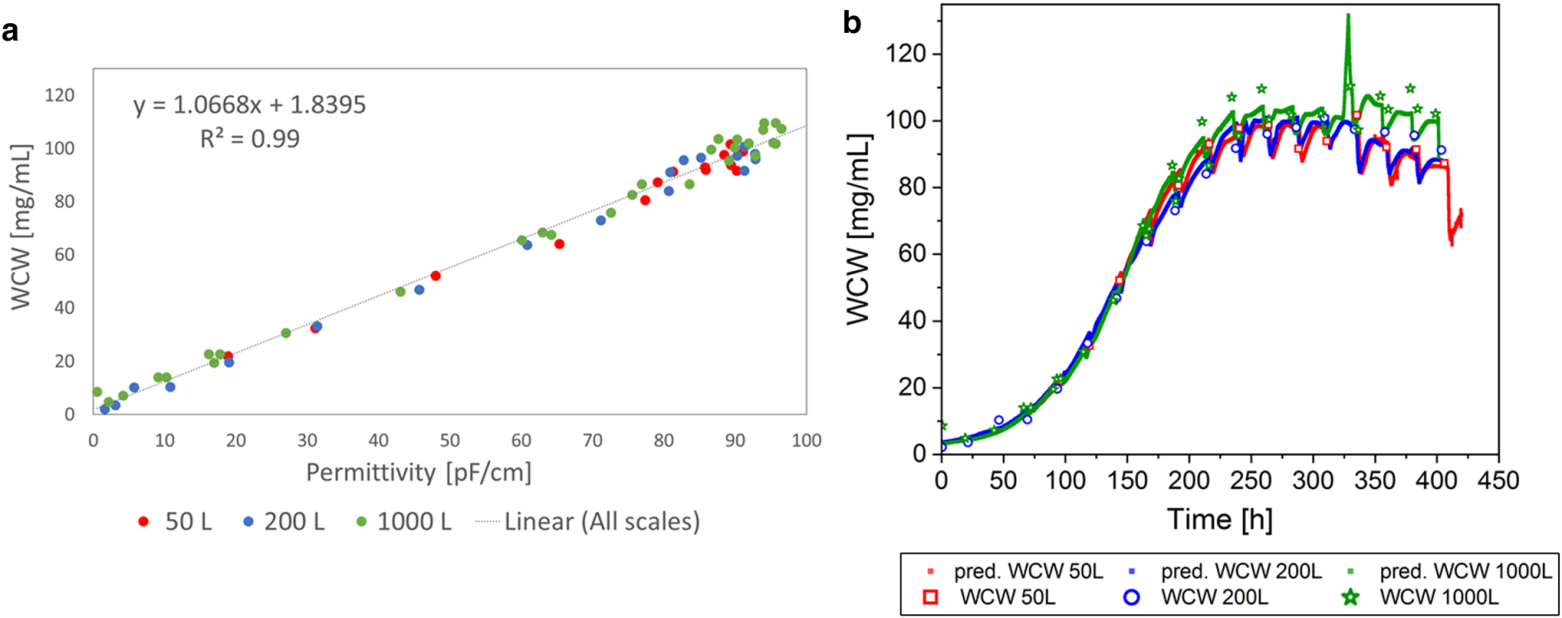
Linear regression model of permittivity and wet cell weight (WCW) for Process B, including all cultivations from 50 L up to 1000 L bioreactor volume (**a**). Predictions (pred.) based on the online permittivity signal for all bioreactor scales using the equation from the linear regression (**b**)

**Fig. 7:**
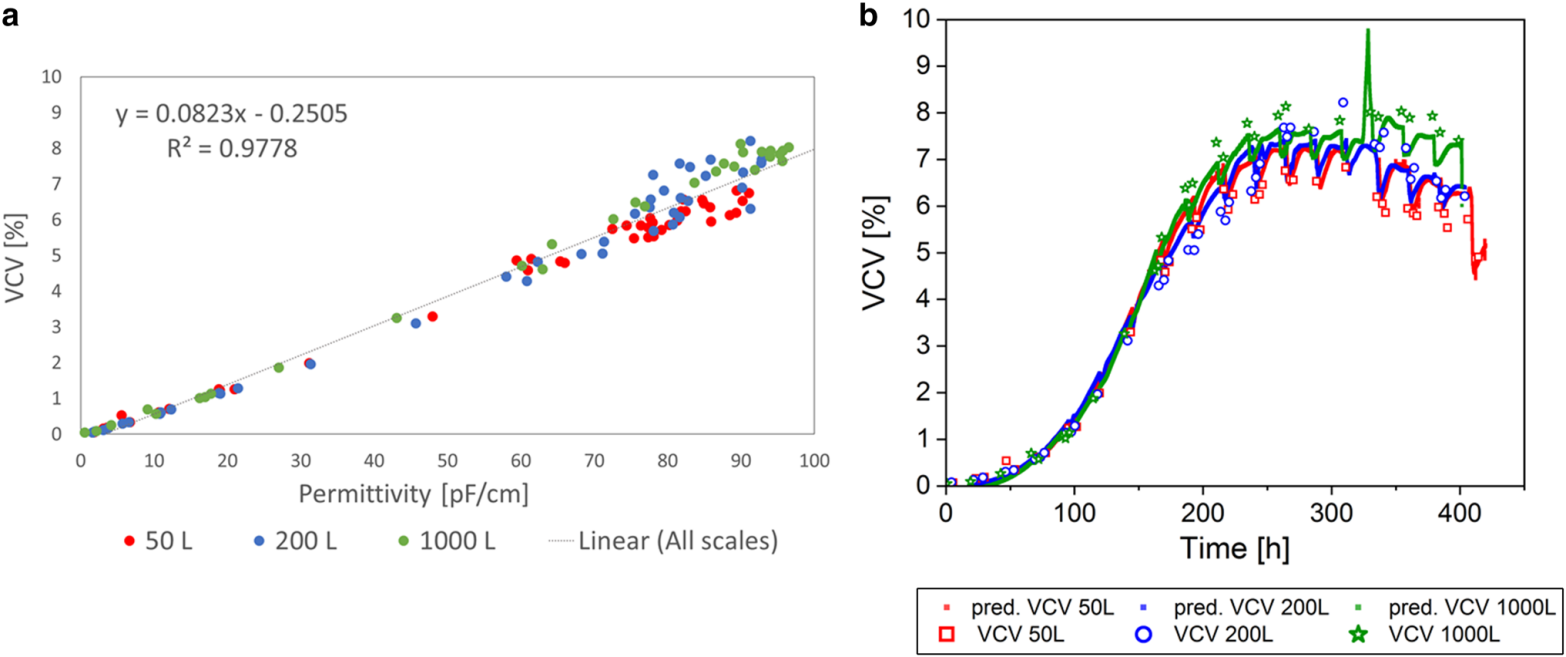
Linear regression model of permittivity and viable cell volume (VCV) for Process B, including all cultivations from 50 L up to 1000 L bioreactor volume (**a**). Predictions (pred.) based on the online permittivity signal for all bioreactor scales using the equation from the linear regression (**b**)

**Fig. 8:**
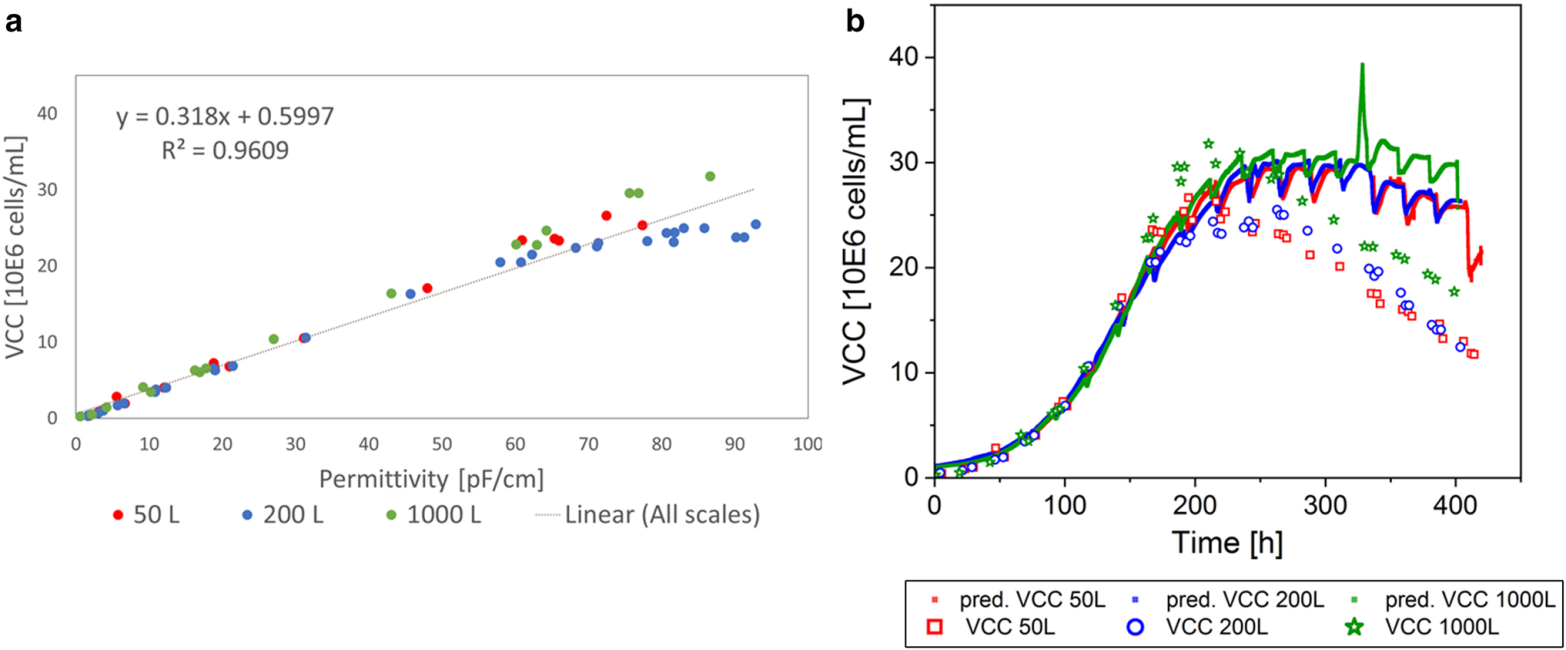
Linear regression model of permittivity and viable cell concentration (VCC) for Process B, including all cultivations from 50 L up to 1000 L bioreactor volume (**a**). VCC only considered up to peak VCC for linear regression. Predictions (pred.) based on the online permittivity signal for all bioreactor scales using the equation from the linear regression (**b**)

To prove the results during scale-up of the single-use bioreactors that are shown in Process A, selected single-use bioreactor scales (50 L, 200 L, and 1000 L) were cultivated and the same parameters were investigated with a different CHO cell culture fed-batch process, here named Process B. The same linear regressions were made for WCW, VCV, and VCC, including all bioreactor scales done for Process B (Figs. 6, 7, 8). As previously shown for Process A, each cultivation for Process B was colored individually within the linear regression model to prove scale independency (Figs. 6a, 7, 8a). The offline data points were well mixed for each correlation and there was no trend for a specific bioreactor scale or cultivation detected. Therefore, it can be concluded that the bioreactor scale itself had no influence on the measurement method and linear regression model.

For Process B, the coefficient of determination for the WCW was drastically higher with 99% (Fig. 4a) compared to 78.6% in Process A (Fig. 6). In contrast to Process A, more data points were incorporated in the correlation, especially at low WCW in the early process phase. Moreover, the scattering of the measurement values was reduced resulting in a regression with a straight line through the origin. The performance of the offline reference method was strongly improved compared to previous results that might be aligned with fewer operators and a more detailed standard operating procedure. The high coefficient of determination shows the potential of correlating permittivity and WCW measurements as it was expected based on the theory of each measurement method. To conclude, the prediction of the WCW based on the online signal is in agreement with the offline data and can be used to describe the WCW accurately during scale-up, as long as the linear regression is based on a representative data set with a high amount of measurement values. Even more process deviations can be detected thanks to the online signal. A peak after 330 h of cultivation was detected in the 1000 L bioreactor for all predicted offline parameters (Figs. 6b, 7, 8b). After verification with the DO measurements of the cultivation, a DO blackout was recognized. The peak in the permittivity signal might be related to a stress response of the cells that increases in diameter rapidly. In addition to the classical DO probe, the online permittivity signal detected the problem immediately and gave information about the cell response and not only the bioreactor environment due to the blackout. The combination of both probes saved the batch, due to fast reaction times and increased process understanding. This example shows that the online capacitance probe is a powerful tool to give insights into cell metabolic states, to control bioprocesses, and to keep the process within an approved trajectory.

The coefficient of determination of the linear regression of the permittivity and the VCV was 97.8% (Fig. 7a). The error of the linear regression was within the range of 16% determined for the offline VCV error (Table1). Therefore, the prediction of the VCV is consistent in all scales and is suitable as online monitoring tool for all bioreactor scales (Fig. 7b).

The VCC in the exponential phase was described with a coefficient of determination of 96.1% (Fig. 8a) and the prediction was suitable for all scales (Fig. 8b). Similar to the case of process A, a clear deviation of the prediction and the offline measurement was detected after the VCC peak was reached. This result was expected based on the described limitations in the measurement method.

The presented results show the scalability of the online bio-capacitance measurement to describe the WCW, the VCV, and the VCC (in the exponential phase) given in all bioreactor scales and for different CHO fed-batch processes. In addition, the online monitoring enabled fast operative steps to keep the process within the pre-defined trajectory. This shows the method´s potential for automated process control to keep a process within the accepted design space during a cultivation.

In a final step, the prediction ability of the online measurement was challenged regarding creating a process-independent model. Therefore, a linear regression was applied using the data from Process A and Process B. To see the process dependency of the model, each model was colored individually (Fig. S2). Thus, each process was clearly distinguished for the WCW and the VCC, especially with prolonged culture time. Process B was cultivated for a longer period than Process A. For VCC correlations, it can be discussed whether in a combined approach, the criteria of incorporating VCC values until peak cell concentration is suitable. The peak cell concentration for Process A took place at an earlier process time (~150 h) compared to Process B (~200 h). Moreover, the high error in the offline reference hamper the exact determination of the peak cell concentration. Comparing the diameter at both process times, a clear difference appeared with the prolonged process time between each peak cell concentration (Fig. S3). As capacitance is influenced by diameter changes, this can have an impact on the VCC result for the common correlation that is supported by the better result for VCV correlations for a common approach (Fig. S3b). Thus, the criteria of which values to include VCC correlations might be changed for a combined approach of both processes. Other criteria can be the inflection point of the VCC, curve, or using a fixed change in diameter as criteria. However, in these studies, a fixed diameter change would increase the coefficient of determination, but each process would remain distinguishable (Fig. S2c). Therefore, with the presented method of single-frequency measurements in this work, it is not recommended to use one model for the two differently presented CHO processes or a process-specific calibration is needed in the beginning of each cultivation.

Table 2 summarizes the results in the presented study. In comparison to the literature values and the measurement errors, the coefficients of determination for the selected KPIs were within comparable and acceptable error ranges. Thus, the linear regression models based on the capacitance sensor integration were suitable for each process and process parameters (WCW, VCV, and VCV) resulting in scale-independent online predictions for each process. Detailed process information was gained with online monitoring in regards to the feed profile. Furthermore, the sensor integration enabled process monitoring with fast reaction times to process failures, indicating strong potential for process control (e.g. feed control or determination of harvest point).

**Table 2:** Summary of results and comparison to the literature

Key performance indicators	*R*^2^ in Process A	*R*^2^ in Process B	Expected error of reference method (%)	Literature comparison for linear correlations with capacitance
Wet cell weight	0.79	0.99	1–25	–
Viable cell volume	0.96	0.98	10	0.99—in exponential growth phase of a mammalian cell line [27] 0.98—in exponential growth phase and 0.75—in stationary phase for viable packed cell volume of a mammalian cell line [26]
Viable cell concentration	0.99	0.96	10	0.96—for a mammalian cell line [26] 0.98—in a perfusion process of a mammalian cell line [16]

## Conclusion

In the presented work, it was shown that the online permittivity signal is capable to describe different KPIs for biomass dynamics during process scale-up of different CHO cell culture processes. The sensor integration was successfully demonstrated for single-use bioreactor scales ranging from 50 L up to 2000 L reactor volume and scale-independence of the method was shown. However, limitations of the measurement method for the presented processes in the stationary growth phase and the death phase based on cell diameter changes of apoptotic cells were detected, confirming previous results from the literature. Nevertheless, the correlations of permittivity with VCC in the exponential growth phase and WCW, respectively, VCV throughout the whole process reached coefficients of determination up to 99% that were comparable to the literature reports and the error of the offline reference methods. The sensor implementation to all bioreactor scales enabled a robust and reliable online monitoring of cell growth in all fed-batch cultures. This can lead to faster process development and mitigation of process risks, and therefore a more robust production process. The presented approach applied in process control can lead to save resources and prevent cultivations from failure by keeping the batch within an approved trajectory.

## Electronic supplementary material

Below is the link to the electronic supplementary material.

Supplementary file1 (DOC 334 kb)

## References

[1] Willoughby N (2006). Scaling up by thinking small: a perspective on the use of scale-down techniques in process design. J Chem Technol Biotechnol.

[2] Xing Z, Kenty BM, Li ZJ (2009). Scale-up analysis for a CHO cell culture process in large-scale bioreactors. Biotechnol Bioeng.

[3] Justice C, Brix A, Freimark D (2011). Process control in cell culture technology using dielectric spectroscopy. Biotechnol Adv.

[4] Wurm FM (2004). Production of recombinant protein therapeutics in cultivated mammalian cells. Nat Biotechnol.

[5] Baldi L, Hacker DL, Adam M (2007). Recombinant protein production by large-scale transient gene expression in mammalian cells: state of the art and future perspectives. Biotechnol Lett.

[6] Butler M (2005). Animal cell cultures: recent achievements and perspectives in the production of biopharmaceuticals. Appl Microbiol Biotechnol.

[7] Hu W-S, Aunins JG (1997). Large-scale mammalian cell culture. Curr Opin Biotechnol.

[8] Sommeregger W, Sissolak B, Kandra K (2017). Quality by control: Towards model predictive control of mammalian cell culture bioprocesses. Biotechnol J.

[9] Rathore AS, Yu M, Yeboah S (2008). Case study and application of process analytical technology (PAT) towards bioprocessing: use of on-line high-performance liquid chromatography (HPLC) for making real-time pooling decisions for process chromatography. Biotechnol Bioeng.

[10] Kourti T (2006). The process analytical technology initiative and multivariate process analysis, monitoring and control. Anal Bioanal Chem.

[11] Dünnebier G, Tups H (2007). FDA PAT initiative—Eine Anwendersicht zu technischen Möglichkeiten und aktueller industrieller Umsetzung. Chem Ing Tec.

[12] Rudolph G, Brückerhoff T, Bluma A (2007). Optische Inline-Messverfahren zur Zellzahl- und Zellgrößenbestimmung in der Bioprozesstechnik. Chem Ing Tec.

[13] Carvell JP, Dowd JE (2006). On-line measurements and control of viable cell density in cell culture manufacturing processes using radio-frequency impedance. Cytotechnology.

[14] Kell BD, Markx GH, Davey CL (1990). Real-time monitoring of cellular biomass: methods and applications. TrAC Trends Anal Chem.

[15] Konstantinov K, Chuppa S, Sajan E (1994). Real-time biomass-concentration monitoring in animal-cell cultures. Trends Biotechnol.

[16] Mercier SM, Rouel PM, Lebrun P (2016). Process analytical technology tools for perfusion cell culture. Eng Life Sci.

[17] Fernandes J, Currie J, Ramer K (2018). Development of capacitance tools: at-line method for assessing biomass of mammalian cell culture and fixed cell calibration standard. Biotechnol J.

[18] Marison I, Hennessy S, Foley R (2013). The choice of suitable online analytical techniques and data processing for monitoring of bioprocesses. Adv Biochem Eng Biotechnol.

[19] Vojinović V, Cabral J, Fonseca LP (2006). Real-time bioprocess monitoring. Sens Actuat B Chem.

[20] Rathore AS (2014). QbD/PAT for bioprocessing: moving from theory to implementation. Curr Opin Chem Eng.

[21] Streefland M, Martens DE, Beuvery EC (2013). Process analytical technology (PAT) tools for the cultivation step in biopharmaceutical production. Eng Life Sci.

[22] Abu-Absi NR, Kenty BM, Cuellar ME (2011). Real time monitoring of multiple parameters in mammalian cell culture bioreactors using an in-line Raman spectroscopy probe. Biotechnol Bioeng.

[23] Harris CM, Todd RW, Bungard SJ (1987). Dielectric permittivity of microbial suspensions at radio frequencies: a novel method for the real-time estimation of microbial biomass. Enzyme Microbial Technol.

[24] Yardley JE, Kell DB, Barrett J (2000). On-line, real-time measurements of cellular biomass using dielectric spectroscopy. Biotechnol Genet Eng Rev.

[25] Cannizzaro C, Gügerli R, Marison I (2003). On-line biomass monitoring of CHO perfusion culture with scanning dielectric spectroscopy. Biotechnol Bioeng.

[26] Opel CF, Li J, Amanullah A (2010). Quantitative modeling of viable cell density, cell size, intracellular conductivity, and membrane capacitance in batch and fed-batch CHO processes using dielectric spectroscopy. Biotechnol Prog.

[27] Downey BJ, Graham LJ, Breit JF (2014). A novel approach for using dielectric spectroscopy to predict viable cell volume (VCV) in early process development. Biotechnol Prog.

[28] Ansorge S, Lanthier S, Transfiguracion J (2011). Monitoring lentiviral vector production kinetics using online permittivity measurements. Biochem Eng J.

[29] Elias CB, Zeiser A, Bédard C (2000). Enhanced growth of Sf-9 cells to a maximum density of 5.2 x 10(7) cells per mL and production of beta-galactosidase at high cell density by fed batch culture. Biotechnol Bioeng.

[30] Ducommun P, Kadouri A, von Stockar U (2002). On-line determination of animal cell concentration in two industrial high-density culture processes by dielectric spectroscopy. Biotechnol Bioeng.

[31] Konakovsky V, Yagtu AC, Clemens C (2015). Universal capacitance model for real-time biomass in cell culture. Sensors (Basel).

[32] Moore B, Sanford R, Zhang A (2019). Case study: the characterization and implementation of dielectric spectroscopy (biocapacitance) for process control in a commercial GMP CHO manufacturing process. Biotechnol Prog.

[33] Ansorge S, Esteban G, Schmid G (2010). On-line monitoring of responses to nutrient feed additions by multi-frequency permittivity measurements in fed-batch cultivations of CHO cells. Cytotechnology.

[34] Párta L, Zalai D, Borbély S (2014). Application of dielectric spectroscopy for monitoring high cell density in monoclonal antibody producing CHO cell cultivations. Bioprocess Biosyst Eng.

[35] Zitzmann J, Weidner T, Eichner G (2018). Dielectric spectroscopy and optical density measurement for the online monitoring and control of recombinant protein production in stably transformed drosophila melanogaster S2 cells. Sensors (Basel).

[36] Eibl R, Kaiser S, Lombriser R (2010). Disposable bioreactors: the current state-of-the-art and recommended applications in biotechnology. Appl Microbiol Biotechnol.

[37] Glindkamp A, Riechers D, Rehbock C (2009). Sensors in disposable bioreactors status and trends. Adv Biochem Eng Biotechnol.

[38] Shukla AA, Gottschalk U (2013). Single-use disposable technologies for biopharmaceutical manufacturing. Trends Biotechnol.

[39] Zhang X, Stettler M, de Sanctis D (2009). Use of orbital shaken disposable bioreactors for mammalian cell cultures from the milliliter-scale to the 1000-liter scale. Adv Biochem Eng Biotechnol.

