# Supplementary material for: Monitoring online biomass with a capacitance sensor during scale-up of industrially relevant CHO cell culture fed-batch processes in single-use bioreactors
Source: Bioprocess Biosyst Eng. 2019 Sep 23;43(2):193–205. doi: 10.1007/s00449-019-02216-4 (PMC6960217; doi:10.1007/s00449-019-02216-4)
Supplement: Supplementary file 1 — Supplementary file1 (DOC 334 kb) [file 449_2019_2216_MOESM1_ESM.doc]

Paper: Monitoring online biomass with a capacitance sensor during scale-up of industrially relevant CHO cell culture fed-batch processes in single-use bioreactors.


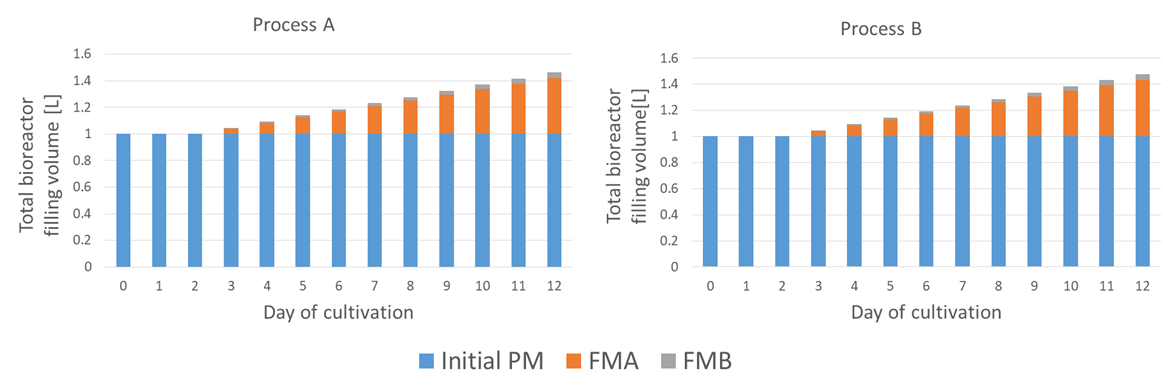


S1: Volume changes inside the bioreactor based on the feed during the two cell culture processes exemplarily shown for a bioreactor with a starting volume of 1 L. The bioreactor is filled with an initial volume of 1 L process medium (PM) and feed medium A (FMA) and feed medium B (FMB) are added starting from day 3 of cultivation.


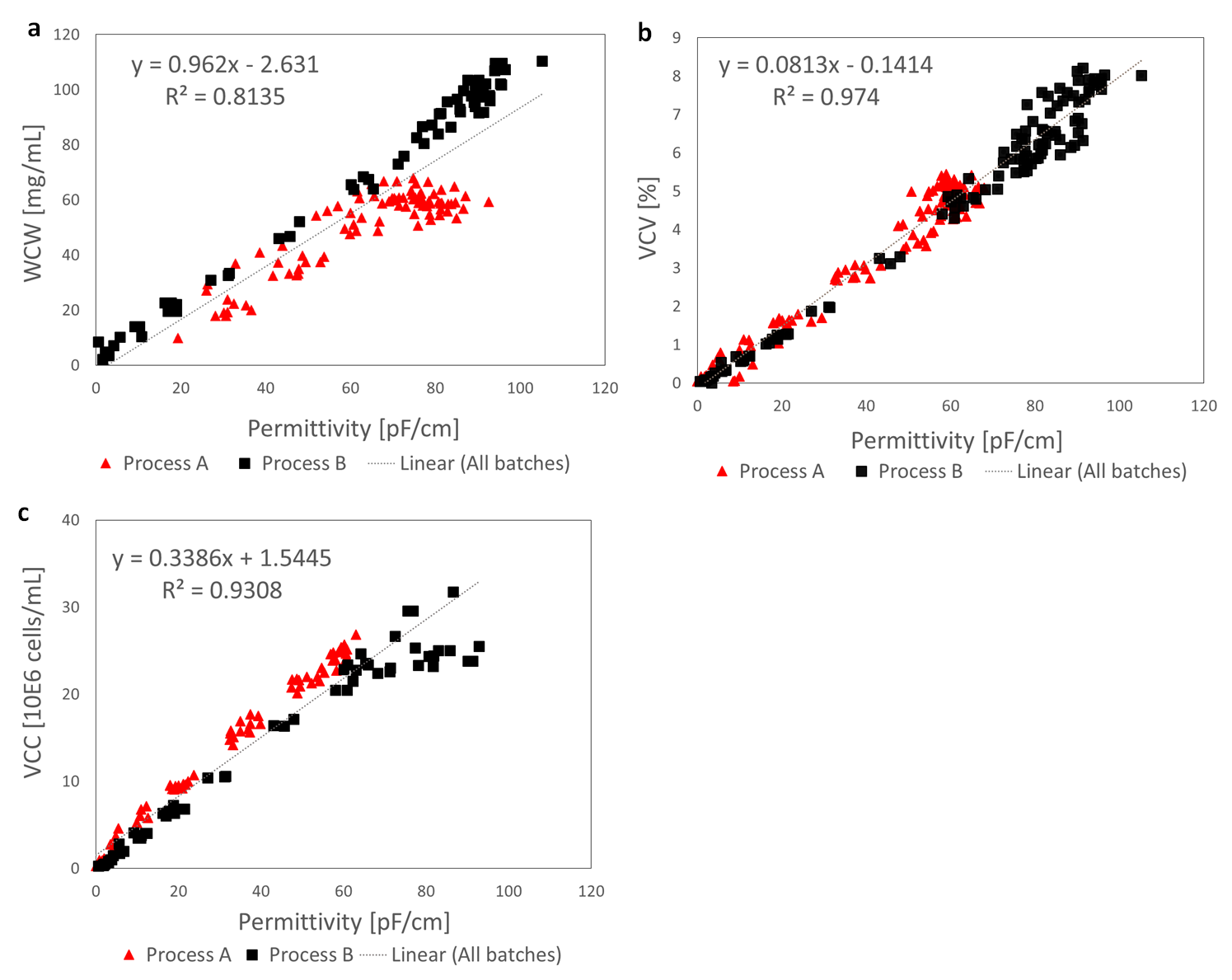


S2: Common linear regression models for Process A and B for each parameter: Wet Cell Weight (A), Viable Cell Volume (B) and the Viable Cell Concentration (VCC) up to peak VCC (C).


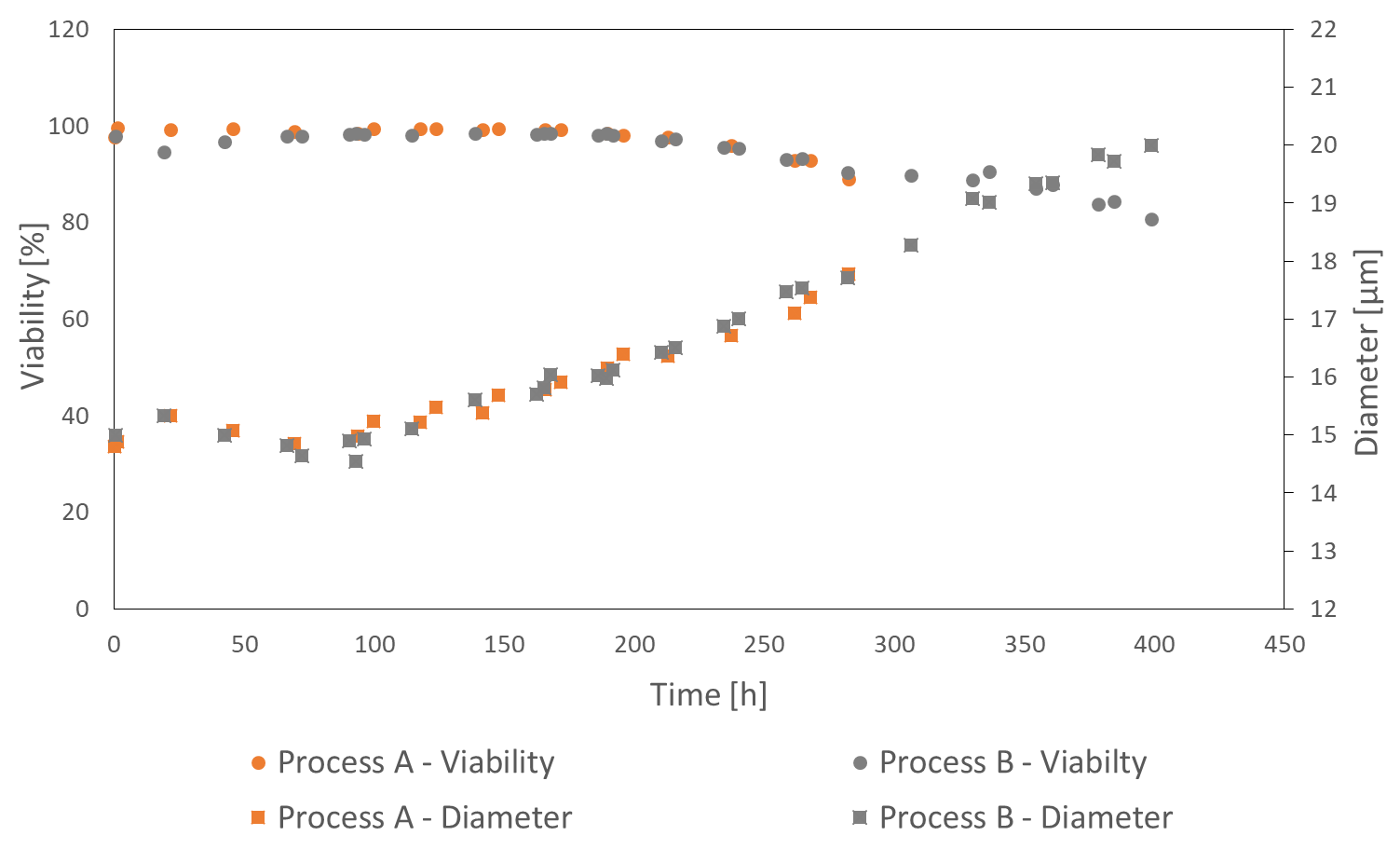


Figure S3: Cell diameter and Viability comparison of 1000 L bioreactors for Process A and Process B.
